# Supplementary material for: Ursolic Acid Enhances the Sensitivity of MCF-7 and MDA-MB-231 Cells to Epirubicin by Modulating the Autophagy Pathway
Source: Molecules. 2022 May 25;27(11):3399. doi: 10.3390/molecules27113399 (PMC9182048; doi:10.3390/molecules27113399)
Supplement: Supplementary file 1 [file molecules-27-03399-s001.zip › Supplementary materials .pdf]

Supplemental Information

Ursolic Acid Enhances the Sensitivity of MCF-7 and MDA-MB-231 Cells to Epirubicin by Modulating the Autophagy Pathway

Zhennan Wang [1,2,†], Pingping Zhang [2,†], Huan Jiang [1], Bing Sun [1], Huaizhi Luo [1] and Aiqun Jia [1,2,*]

[1] School of Environmental and Biological Engineering, Nanjing University of Science and Technology, Nanjing 210094, China; 15050525636@163.com (Z.W.); huanjiang929@foxmail.com (H.J.); yangwenleg@163.com (B.S.); rohizer@163.com (H.L.)

[2] State Key Laboratory of Marine Resource Utilization in South China Sea, School of Pharmaceutical Sciences, Hainan University, Haikou 570228, China; bbsuju2@163.com

* Correspondence: ajia@hainanu.edu.cn; Tel.: +86-898-66254967; Fax: +86-898-66254967

† These authors contributed equally to this work.

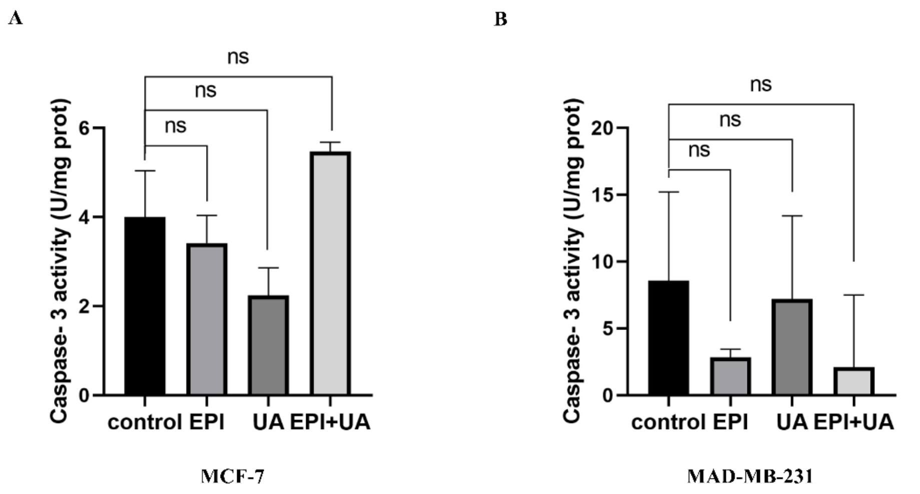

Figure S1. ELISA analysis of the caspase3 activity in MCF-7 (A) and MAD-MB-231 (B) cells. * P<0.05, ** P<0.01, *** P<0.005.

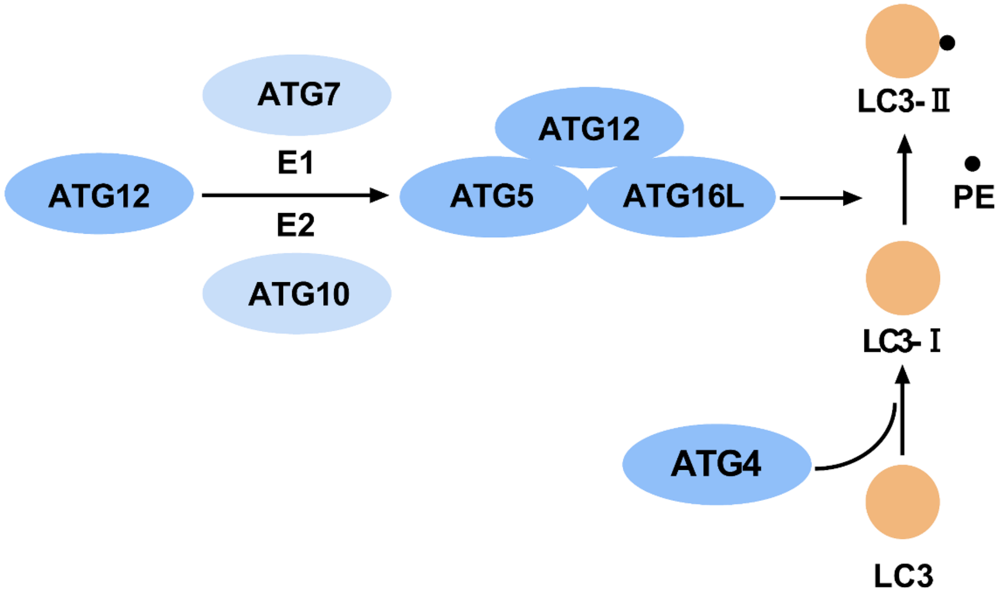

Figure S2. LC3-II protein assembly process.
